# The lamina-spinous process-ligament complex implantation for the spinal stability: A case report and review of literature

**DOI:** 10.1097/MD.0000000000042044

**Published:** 2025-04-04

**Authors:** Tao Lu, Huichun Yuan, Xueqin Hu, Wuyang Cao, Zeng Yang, Zhiwei Zhou, Haibin Leng, Nianhua Wang, Lixin Xu

**Affiliations:** a Department of Neurosurgery, Changde Hospital, Xiangya School of Medicine, Central South University (The first people’s hospital of Changde city), Changde, China.

**Keywords:** cervicothoracic fusion, intramedullary tumor, lamina, ligament complex implantation, spinous process

## Abstract

**Rationale:**

The posterior cervicothoracic fusion is a common surgical skill for the treatment of spinal diseases. Since postoperative complications contribute to compressive neurological symptoms, this case is reported to provide a surgical option for the preservation of spine stability and bone fusion.

**Patient concerns:**

A 36-year-old male presented numbness of both lower limbs for more than half a year, accompanied by unstable walking for more than 2 months.

**Diagnoses:**

Clinical examination revealed grade 4 muscle strength of both lower extremities, low muscle tension, loss of abdominal wall reflex, cremasteric reflex and anal reflex, and unsteady gait. Magnetic resonance imaging suggested a 5th cervical vertebrae-2nd thoracic vertebrae (C5-T2) intramedullary space-occupying mass.

**Interventions:**

Written informed consent was obtained from the patient before surgery. To relieve his symptom and preserve spine stability, we performed an operation to remove the C5-T2 intramedullary ependymoma. Meantime, the laminae-spinous process complex was replanted and fixed in situ.

**Outcomes:**

Magnetic resonance imaging indicated that the tumor was completely resected. There are no delayed bleeding and instances of implant loosening. There were no tumor recurrence, spinal stenosis, spinal instability, and spondylolisthesis, and part of vertebral laminae have formed bony healing.

**Lessons:**

Our case suggests that the in situ preservation of supraspinous ligament longitudinal continuity of lamina-spinous process-ligament complex implantation skill will provide a surgical option for the preservation of spine stability and bone fusion in patients with long-segment intraspinal tumors.

## 1. Introduction

Posterior cervicothoracic fusion is a common surgical skill for the treatment of intervertebral disc herniation, intraspinal canal tumors and other diseases.^[1–3]^ Since the cervicothoracic vertebral body has a large range of motion, the possibility of degeneration of adjacent vertebral segments is high, which is easy to cause the spinal instability after surgery. Previous studies have reported that increased pressure at the cervicothoracic junction caused higher stress in adjacent intervertebral discs, and a significant increase in the incidence of revision surgery in multi-segment spinal structures involving the 7th cervical vertebrae.^[4]^ Resection of long-segment intraspinal tumors involves the reconstruction of the stability of the cervicothoracic vertebrae, which is considered a difficult point in surgical treatment.^[5,6]^ At present, after resection of the posterior structure of the cervicothoracic vertebrae to expose the spinal canal and resection of spinal canal tumors, there are 2 ways to treat the cervicothoracic vertebrae, 1 is not to perform internal fixation and fusion^[7,8]^ and another is to perform posterior vertebral internal fixation and fusion.^[9–11]^ The former way destroys the original anatomical structure of the cervical and thoracic spine, which increased possibility of vertebral instability and deformity; The latter loses the range of motion, resulting in limited motion of the cervical spine. The laminar implantation technology is used to treat spinal canal tumors to restore the original structure of the spine and rebuild spinal stability, but it is mostly applied to thoracic and lumbar spinal canal tumors, and this technology destroys the continuity of the supraspinous ligament.^[12,13]^ Since 2014, we have used laminar implantation technique to preserve the continuity of the supraspinous ligament to treat benign tumors in the cervicothoracic spinal canal. This article reports clinical data of a patient with long-segment (C5-T2, 5 segments) cervicothoracic ependymoma. The report is as follows.

## 2. Case report

**Figure 1.**
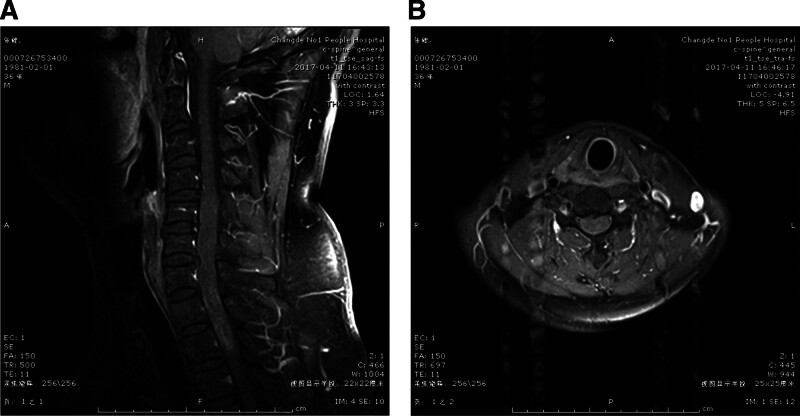
Diagnostic MRI images for the intramedullary ependymoma (C5-to-T2, 5 segments). (A, B) The sagittal and axial T1W enhanced MRI images reveal the intramedullary space-occupying mass in the cervical 5 to thoracic 2 spinal cords, which shows uniform moderate strengthening signal and cavities. C5 = 5th cervical vertebrae, MRI = magnetic resonance imaging, T2 = 2nd thoracic vertebrae.

A 36-year-old male patient presented with numbness of both lower limbs over the period of half a year, accompanied by gait instability for more than 2 months. Physical examination: normal bilateral gag reflex, normal muscle strength of both upper limbs and muscular tension, hyperreflexia of bilateral biceps/triceps, periosteum, and knee jerk, ankle clonus (++), grade 4 muscle strength of both lower extremities, normal muscular tension, loss of abdominal reflexes, loss of cremasteric reflex and anal reflex, gait instability, negative Babinski sign. MRI suggested intraspinal occupying lesion of C5-T2: ependymoma? (Fig. 1).

**Figure 2.**
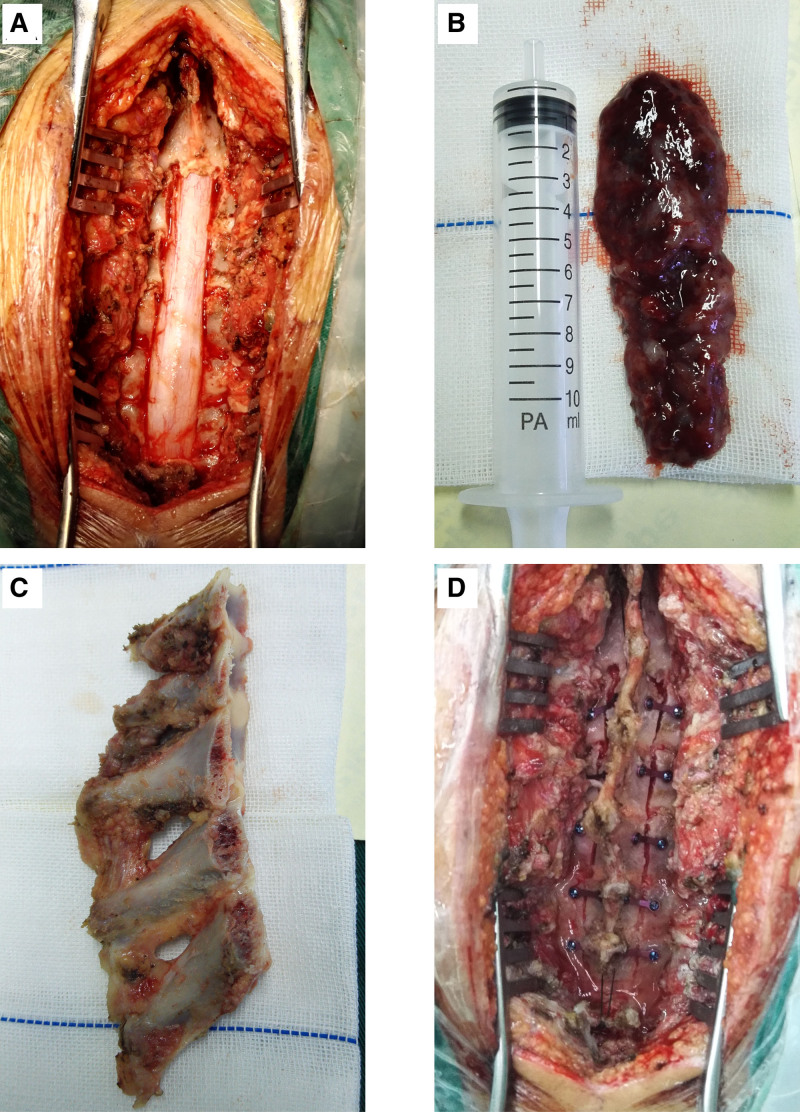
The lamina-spinous process-ligament complex implantation skill for en bloc tumor excision. (A) The exposure of spinal cord at C5-to-T2 levels. (B) The tissue of whole intramedullary ependymoma was totally removed. (C) The intraoperative image of the lamina-spinous process-ligament complex. (D) The titanium plates and titanium nails were used for the lamina-spinous process-ligament complex reduction during the operation. C5 = 5th cervical vertebrae, T2 = 2nd thoracic vertebrae.

Operation: we initially exposed the C5-T2 laminae and made the C5-T2 spinous laminae complex using the milling cutter. After exposure, we observed high dural tension, then, opened the dura longitudinally and separated the spinal cord along the posterior median sulcus. The intramedullary space-occupying mass presented slightly dark red covered with capsule. We continuously separated the tumor block along the periphery. The spinal cord was protected under a neurophysiological monitoring. Finally, the whole tumor was resected and its size was about 1.5*1.5*11 cm. After the dura was sutured, 10 connecting straps and 20 screws were used to fix the laminae and spinous process complex, then, the spinous process and supraspinous ligament were sutured. After operation, he was further treated at neurosurgery intensive care unit (Fig. 2).

### 2.1. Pathologic result

(C5-T2) ependymoma. Immunohistochemistry: GFAP (+), EMA (−), S-100 (+), Vimentin (+), CK (−), Ki-67 (+, 3%).

### 2.2. Postoperative changes

After treatment at neurosurgery intensive care unit for 1.5 days, he was transferred to the general ward for continuing treatment. Prophylactic cefuroxime was used as anti-infective therapy for 2 days. Tramadol was given for 3 days after operation. The follow-up computed tomography showed no delayed bleeding and the position of the connecting straps and screws was appropriate. MRI suggested that tumor was completely resected and the spinal canal was adequately decompressed. On the third day after operation, he reported that the pain was significantly relieved, the grip strength and sensation of both upper limbs were influenced, and the numbness was obvious. Thus, the hormone and methylcobalamin were given to protect nerve function. One month post operation, he could finish appropriate activities with the assistance of external fixator. For improve sensation function, methylcobalamin was given until numbness disappeared.

**Figure 3.**
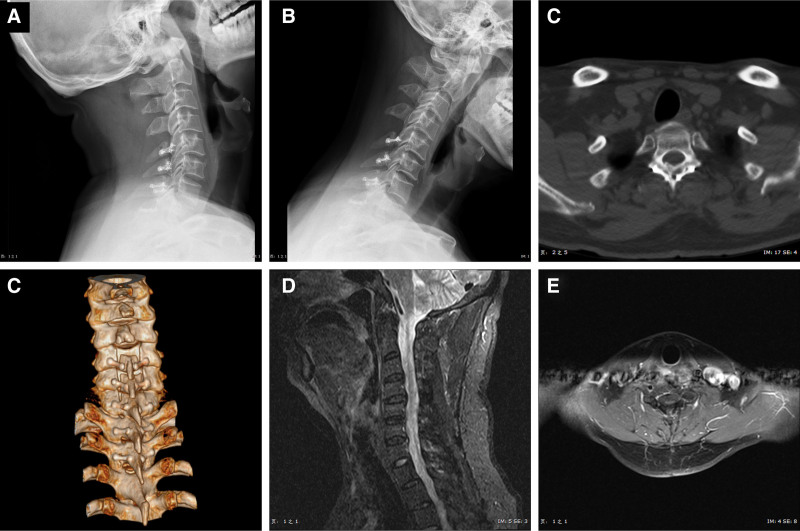
Follow-up 4 yr after surgery. (A, B) The dynamic X-ray examination verified that the spinal stability was preserved. (C, D) The CT scan showed that the bone fusion happened within the lamina-spinous process-ligament complex. (D, E) The sagittal and axial enhanced MRI images showed the recurrency by tumor did not happen. CT = computed tomography, MRI = magnetic resonance imaging.

After 4 years of follow-up, there were no tumor recurrence, no spinal stenosis, no spinal instability and spondylolisthesis, and part of vertebral laminae have been bony healing (Fig. 3).

## 3. Discussion

Intramedullary ependymoma is one of the most common intraspinal tumor, which originating from the central ependymal cells of the spinal cord, and highly occurring in the cervical and thoracic cords. The ependymoma grew rostral and caudal in the central canal, which compressing the spinal cord and causing neurological dysfunction. Until now, resection is the most effective treatment. With the improvement of microsurgery skills, the implementation of minimally invasive concepts, and the application of neuroelectrophysiological monitoring techniques, the rate of total tumor resection has been significantly increased, and the surgical risk has been obviously reduced.^[14,15]^

Long-segment ependymoma should be fully exposed during operation, which requires incision of multiple segmental laminae. This exposure process may affect spinal stability and result in posterior process deformity and iatrogenic spinal cord compression injury. The importance of postoperative stability reconstruction is increasingly emphasized. Yu et al^[16]^reported that for patients with more than 2 articular process destruction or resection, the cervical vertebrae should be fixed and fused for safety consideration. Simultaneous removal of cervical laminae, interspinous, and supraspinous ligaments has a greater impact on spinal stability and usually requires internal fixation to maintain spinal stability.^[17]^ Therefore, it is important to choose a reasonable surgical method and stability reconstruction method for the treatment of intraspinal tumors at the junction of the cervical and thoracic cords, which not only clearly exposes the spinal canal to completely remove the intraspinal tumor, but also protects the normal anatomical structure of the spine. The unilateral hemilamectomy and unilateral open-door laminoplasty were used to expose the spinal canal and resect the tumor mass, which has the advantage of effectively reducing damage to posterior structure of the spine, but there are shortcomings of insufficient exposure and difficulty in resection for patients with a large range of intraspinal tumor.^[18–20]^ Laminar repositioning has the advantages of remodeling anatomy, small damage, and low incidence of postoperative complications. After the resection of the cervicothoracic junction lesion, due to the large joint activity, the pedicle screw-rod system is mostly used to maintain spinal stability, and the follow-up time is generally 12 months. However, the follow-up time of spinal function is not enough to reflect the changes in spinal stability, and long-term observation is still needed in the future. In addition, the screw-rod is prone to generate large artifacts during follow-up MRI examination, which affect the recognition of normal spine and spinal cord structures after operation. In our case, the laminectomy exposed to the lateral side of the spinal canal is enough, without exposing the lateral facet joint, and protect the lateral facet joint. When separating multi-segmental laminae, we separated the supraspinous ligament from the spinous-supraspinous ligament complex, and preserved the integrity of the supraspinous ligament in situ. To reduce bone defects, we further used a milling cutter or a small grinding drill to open the laminae. After the intraspinal tumor was removed, the laminae-spinous process complex was replanted and fixed in situ, and the attached muscle and supraspinous ligament were reconstructed, and the external fixation device was worn for 1 month after operation. Through more than 6 years of follow-up, we found that the overall mobility and stability of the cervical and thoracic vertebrae were appropriate, and the cervical dynamic X-ray and computed tomography suggested vertebral stability and laminar bony healing, without adjacent spondylosis.

Advantages of in situ preservation of supraspinous ligament continuity of lamina-spinous process-ligament complex implantation skill for the treatment of benign tumors in the long-segment cervical and thoracic spinal canal: In situ preservation of supraspinous ligament continuity, after separation of the supraspinous ligament, the cervical and thoracic vertebral laminae, spinous process and interspinous ligament complex were removed, the spinal canal was exposed, the tumor was resected in situ. Then, the cervical and thoracic vertebral laminae, attached muscle tissue and supraspinous ligament were reconstructed. Thus, there is no need for vertebral body fusion. The integrity of the anterior and middle columns of the cervical and thoracic vertebrae was preserved, while the integrity of the muscular ligament attachment point, supraspinous and interspinous ligaments is preserved, which were most important for stability in the posterior column. The operation preserved the integrity of the entire supraspinous ligament and the interspinous ligament in situ, preserved part of the blood supply, and was conducive to the healing of the laminae. The cervical and thoracic lamina-spinous-interspinous ligament complex with complete implantation provided a bony attachment point for the paravertebral muscle, and the muscles on the spinous process and the supraspinous ligament attachment point on the spinous process of the cervical and thoracic vertebra were reconstructed by suturing and fixing the muscle, supraspinous ligament and the tail end of the spinous process, and the bony structure of the spinal canal was also remodeled, which can prevent epidural fibrosis and long-term degenerative deformity instability.

Disadvantages: Due to the in situ replantation of the cervical and thoracic lamina-spinous process-ligament complex, the spinal canal was not enlarged and decompressed. If the intraspinal tumor recurs, it can induce spinal nerve compression symptoms in the early stage. Therefore, the contraindications to this operation are suspected intraspinal malignancy, intraspinal tumors invaded the lamina, and intraspinal tumors that cannot be completely resected. The indications for this operation are patients with benign tumors in the long-segment spinal canal such as the junction of the cervical and thoracic segments.

## 4. Conclusion

The in situ replantation technique of lamina-spinous process-ligament complex with in situ preservation of the continuity of the supraspinous ligament was used to treat benign tumors in the spinal canal in the cervicothoracic junction area, which could reconstruct the normal anatomy of the posterior column of the spine and better maintain the range of motion and stability of the cervical spine. However, as case report, the number of cases is small and no control group has been set up for comparative study, and a large-sample randomized controlled study is needed to further verify the accuracy of the conclusions.

## Author contributions

**Conceptualization:** Nianhua Wang, Lixin Xu.

**Data curation:** Tao Lu, Huichun Yuan.

**Funding acquisition:** Tao Lu.

**Methodology:** Xueqin Hu, Wuyang Cao, Zeng Yang, Zhiwei Zhou, Haibin Leng.

**Resources:** Xueqin Hu, Wuyang Cao, Zeng Yang, Zhiwei Zhou, Haibin Leng.

**Supervision:** Nianhua Wang, Lixin Xu.

**Validation:** Xueqin Hu, Wuyang Cao, Zeng Yang, Zhiwei Zhou, Haibin Leng, Nianhua Wang, Lixin Xu.

**Writing – original draft:** Tao Lu, Huichun Yuan, Nianhua Wang, Lixin Xu.

**Writing – review & editing:** Tao Lu, Huichun Yuan, Nianhua Wang, Lixin Xu.

## References

[1] Xia LL Tang J Huang SL. Primary intraspinal benign tumors treated surgically: an analysis from China. Br J Neurosurg. 2021;35:603–6. 34085892 10.1080/02688697.2021.1923648

[2] Patel P Mehendiratta D Bhambhu V Dalvie S. Clinical outcome of intradural extramedullary spinal cord tumors: a single-center retrospective analytical study. Surg Neurol Int. 2021;12:145. 33948315 10.25259/SNI_839_2020 PMC8088529

[3] Zabsonre SD Bambara AT Ouattara S. Histological profile and progression of intraspinal tumors after surgery. Pan Afr Med J. 2021;38:128. 33912298 10.11604/pamj.2021.38.128.21214 PMC8051254

[4] Gelfand Y Franco D Kinon MD. Selecting the lowest instrumented vertebra in a multilevel posterior cervical fusion across the cervicothoracic junction: a biomechanical investigation. J Neurosurg Spine. 2023;38:389–95. 36681959 10.3171/2022.10.SPINE22381

[5] Kaya RA Türkmenoğlu ON Koç ON. A perspective for the selection of surgical approaches in patients with upper thoracic and cervicothoracic junction instabilities. Surg Neurol. 2006;65:454–63; discussion 463. 16630904 10.1016/j.surneu.2005.08.017

[6] Yang X Wan W Gong H Xiao J. Application of individualized 3D-printed artificial vertebral body for cervicothoracic reconstruction in a six-level recurrent chordoma. Turk Neurosurg. 2020;30:149–55. 31049920 10.5137/1019-5149.JTN.25296-18.2

[7] Tarabay B Gennari A Boubez G Wang Z Shedid D Yuh SJ. Minimally invasive approach for complete resection of a cervical intramedullary tumor via a dorsal root entry zone using fixed tubular retractor. Cureus. 2022;14:e28457. 36185933 10.7759/cureus.28457 PMC9514149

[8] Thavara BD Kidangan GS Rajagopalawarrier B. Analysis of the surgical technique and outcome of the thoracic and lumbar intradural spinal tumor excision using minimally invasive tubular retractor system. Asian J Neurosurg. 2019;14:453–60. 31143261 10.4103/ajns.AJNS_254_18 PMC6516036

[9] Guo X Yang S Li Z Yang D Ding W. Clinical effect of laminectomy with lateral mass screw fixation in treating cervical schwannoma: a retrospective study. Biomed Res Int. 2022;2022:8512374. 35528181 10.1155/2022/8512374 PMC9076331

[10] Wang H Huo Y Li L. Clinical efficacy of laminectomy with instrumented fixation in treating thoracolumbar intradural extramedullary schwannomas: a comparative study. Med Sci Monit. 2020;26:e921719. 32515362 10.12659/MSM.921719 PMC7299065

[11] Yoon ST Hashimoto RE Raich A Shaffrey CI Rhee JM Riew KD. Outcomes after laminoplasty compared with laminectomy and fusion in patients with cervical myelopathy: a systematic review. Spine. 2013;38(22 Suppl 1):S183–194. 23963000 10.1097/BRS.0b013e3182a7eb7c

[12] Duan Y Ma J Miao S Zhang J Deng J Wu H. Comparison of total laminectomy and pedicle screw internal fixation with ultrasonic- and microscopic-assisted laminectomy replantation for tumors of the lumbar spinal canal: a retrospective study of 60 cases from a single center. Med Sci Monit. 2021;27:e931768. 34548468 10.12659/MSM.931768 PMC8475735

[13] Papagelopoulos PJ Peterson HA Ebersold MJ Emmanuel PR Choudhury SN Quast LM. Spinal column deformity and instability after lumbar or thoracolumbar laminectomy for intraspinal tumors in children and young adults. Spine. 1997;22:442–51. 9055374 10.1097/00007632-199702150-00019

[14] Kaya RA. Surgical excition of spinal intradural meningiomas through a single-sided minimally invasive approach: key-hole laminotomy. Asian Spine J. 2015;9:225–31. 25901234 10.4184/asj.2015.9.2.225 PMC4404537

[15] Massimi L Battaglia D Paternoster G Martinelli D Sturiale C Di Rocco C. Segmental spinal myoclonus and metastatic cervical ganglioglioma: an unusual association. J Child Neurol. 2009;24:365–9. 19258299 10.1177/0883073808323027

[16] Yu Y Hu F Zhang X Gu Y Xie T Ge J. Application of the hemi-semi-laminectomy approach in the microsurgical treatment of C2 schwannomas. J Spinal Disord Tech. 2014;27:E199–204. 23732182 10.1097/BSD.0b013e318299f606

[17] Li C Ye Y Gu Y Dong J. Minimally invasive resection of extradural dumbbell tumors of thoracic spine: surgical techniques and literature review. Eur Spine J. 2016;25:4108–15. 27371333 10.1007/s00586-016-4677-z

[18] Dobran M Paracino R Nasi D. Laminectomy versus unilateral hemilaminectomy for the removal of intraspinal schwannoma: experience of a single institution and review of literature. J Neurol Surg A Cent Eur Neurosurg. 2021;82:552–5. 33845505 10.1055/s-0041-1722968

[19] Wang ZC Li SZ Qu XF. Application of open-door laminoplasty with ARCH plate fixation in cervical intraspinal tumors. BMC Surg. 2021;21:141. 33740933 10.1186/s12893-021-01140-3 PMC7980540

[20] Lei D Zhou Y Yao D. Efficacy of unilateral hemilaminectomy for intraspinal tumor resection: a systematic review and meta-analysis. Ann Palliat Med. 2021;10:984–99. 32954745 10.21037/apm-20-499

